# Usefulness of real-time PCR for urogenital schistosomiasis diagnosis in preschool children in a high-prevalence area in Angola

**DOI:** 10.1371/journal.pntd.0012384

**Published:** 2024-08-14

**Authors:** Alejandro Mediavilla, Aroa Silgado, Raquel Sánchez-Marqués, Cristina Bocanegra, Arlette Nindia, Fernando Salvador, Zeferino Pintar, Patricia Martínez-Vallejo, Carles Rubio Maturana, Lidia Goterris, Joan Martínez-Campreciós, Sandra Aixut, Inés Oliveira-Souto, María Luisa Aznar-Ruiz-de-Alegría, María Espiau, Israel Molina, Elena Sulleiro

**Affiliations:** 1 Microbiology Department, Vall d’Hebron University Hospital, PROSICS Barcelona, Barcelona, Spain; 2 Universitat Autònoma de Barcelona (UAB), Barcelona, Spain; 3 Centro de Investigación Biomédica en Red de Enfermedades Infecciosas (CIBERINFEC), Instituto de Salud Carlos III, Madrid, Spain; 4 Parasitology Department, Pharmacy Faculty, University of Valencia, Valencia, Spain; 5 International Health Unit Vall d’Hebron-Drassanes, Infectious Diseases Department, Vall d’Hebron University Hospital, PROSICS Barcelona, Barcelona, Spain; 6 Hospital Nossa Senhora da Paz. Cubal, Benguela, Angola; 7 Pediatric Infectious Diseases and Immunodeficiencies Unit, Vall d’Hebron University Hospital, PROSICS Barcelona, Barcelona, Spain; University of Agricultural Sciences and Veterinary Medicine Cluj-Napoca, Life Science Institute, ROMANIA

## Abstract

**Background:**

Urogenital schistosomiasis caused by *Schistosoma haematobium* is highly endemic in the municipality of Cubal in Angola. Currently, diagnosis is based on the observation of *S*. *haematobium* eggs in urine samples by microscopy but this method has low sensitivity. Few studies have been performed using molecular techniques in high-prevalence areas for the detection of *S*. *haematobium*. The objective of this study is to evaluate the usefulness of real-time PCR as a diagnostic technique for urogenital schistosomiasis among preschool-age children and its correlation with morbidity data.

**Methods:**

A cross-sectional study was conducted in Cubal, Angola, involving 97 urine samples from preschool-age children analyzed by the dipstick test, microscopic examination of filtered urine, and real-time PCR. The diagnosis of urogenital schistosomiasis was based on microscopy and/or real-time PCR results. Clinical and ultrasonography evaluation was performed to rule out complications of schistosomiasis.

**Results:**

We detected a total of 64.95% of samples positive by real-time PCR and 37.11% by microscopy. The sensitivity of parasitological diagnosis of urogenital schistosomiasis by real-time PCR and microscopy was 95.45% and 54.55%, respectively, and the sensitivity of real-time PCR compared with microscopy was 91.67%. A positive real-time PCR result was significantly related to older age (mean = 3.22 years), detection of eggs by microscopy, and abnormal urine dipstick results (18.56% with proteinuria, 31.96% with leukocyturia, and 31.96% with microhematuria) (*p*-value<0.05). Ultrasound analysis showed that 23.94% of children had urinary tract abnormalities, and it was significantly related to the real-time PCR diagnosis (*p*-value<0.05).

**Conclusions:**

Real-time PCR is a more sensitive technique than microscopy for urinary schistosomiasis diagnosis in preschool-age children in Cubal. This increase in sensitivity would allow earlier diagnosis and treatment, thus reducing the morbidity associated with schistosomiasis in its early stages.

## Author summary

Schistosomiasis is a neglected tropical disease that causes approximately 200,000 deaths worldwide each year. Urogenital schistosomiasis is caused by *Schistosoma haematobium*, which ultimately leads to lesions in the urinary tract due to egg deposition by the parasite. The World Health Organization recommends regular mass administration of praziquantel to prevent chronicity of the disease in high-endemicity areas. Preschool children are one of the most vulnerable groups, however, only children over two years of age are included in these strategies. The diagnostic techniques commonly used in endemic areas for egg detection by microscopy and urine hematuria have major limitations due to their low sensitivity. These limitations lead to misdiagnosis and undetected infections may eventually lead to the chronic form of the disease. Real-time PCR and microscopy were used to diagnose urogenital schistosomiasis in preschool children in Cubal, a high-transmission municipality in Angola. Urinary tract lesions were assessed by ultrasound. Of the samples tested, 64.95% were positive by real-time PCR, compared with 37.11% by microscopy, indicating sensitivities of 95.45% and 54.55%, respectively. Ultrasound analysis showed urinary tract alterations in 23.94% of the children. The results suggest that transmission of urogenital schistosomiasis in preschool children in Cubal is higher than previously estimated.

## Introduction

Schistosomiasis, or bilharziasis, is a parasitic disease caused by Platyhelminthes belonging to the genus *Schistosoma* [1]. It is estimated that, after malaria, schistosomiasis is the second parasitic disease that causes the greatest morbidity worldwide, with approximately 230–250 million people infected and more than 200,000 deaths annually, 90% of which occur in sub-Saharan African countries [2–4]. In humans, there are two distinct forms of the disease depending on the final location of the adult worms: intestinal and urogenital schistosomiasis (UGS). Intestinal schistosomiasis is caused by *Schistosoma mansoni*, *Schistosoma japonicum*, *Schistosoma mekongi*, *Schistosoma guineensis*, and *Schistosoma intercalatum*. In contrast, UGS is caused by *Schistosoma haematobium* infections [4,5].

Most people with UGS become infected in early life through exposure to water sources infested with *S*. *haematobium* larval cercariae [6]. After penetration of the cercariae through the skin, the worms move to the schistosomula stage and, via the circulatory system, migrate to the liver. Here they transform into adult worms that then move to the vesical venous plexus, where they mate [7]. During this migratory route is when the acute phase of the disease begins, in which the host presents a characteristic symptomatology called Katayama syndrome, including fever, headache, fatigue, abdominal pain, malaise, and urticarial rash, among others [8]. However, in most infected individuals there are no symptoms or they are mildly asymptomatic. If untreated, the disease can enter a chronic phase due to the continuous deposition of eggs, causing a granulomatous response that will lead to inflammation with fibrosis and, occasionally, more severe lesions in the urinary tract (UT) and genitalia, or bladder cancer [8].

The distribution of UGS is restricted to African and Middle Eastern countries [9]. Angola is located on the west coast of Africa and has a population of about 40 million, of which approximately 47% are <15 years old and 20% <5 years old [10]. Benguela is the Angolan region with the second-highest prevalence of schistosomiasis [11]. Cubal is a municipality of Benguela with a high prevalence of UGS detected in previous studies performed in school-aged children (SAC) (61.2%). This prevalence is higher than the national average due to the proximity to water sources, such as the Cubal River, and the increase in screening in this area of Angola [2]. In addition, another study concluded that 85.3% of SACs suffering from UGS showed pathological affectations observed by ultrasound, such as abnormal bladder morphology and thickening of the bladder wall, among the main alterations [12].

Preschool-age children (PSAC) are one of the least studied populations for *S*. *haematobium* infection. Currently, chemoprophylaxis based on praziquantel mass drug administration (MDA) campaigns is the method used to prevent UGS-associated morbidity. Historically, PSACs have been excluded from MDA campaigns due to misinformation about the prevalence of the disease in this age group and the lack of a pediatric formulation of praziquantel. However, starting in 2022, the World Health Organization (WHO) recommends the inclusion of PSAC older than two years in MDA campaigns in areas where the prevalence is >10% [3].

Diagnostic methods for detecting *S*. *haematobium* infection in endemic areas include indirect diagnoses, such as the observation of macrohematuria, or detection of microhematuria, leukocyturia, and/or proteinuria by rapid tests, such as the urine dipstick test (UDT). The use of UDT allows an indirect simple and economical diagnosis of infection; however, in nonendemic areas, the use of this indirect method is not recommended because of its low specificity [13,14].

Egg detection by urine microscopy is the gold standard method for the diagnosis of UGS [15]. However, this technique presents some limitations: low sensitivity (particularly in patients with low parasite density, and mild or asymptomatic infections), it is time-consuming, and is affected by the daily variability in egg excretion. These technical limitations may result in a large number of cases going undetected and untreated. These asymptomatic individuals with very low parasite density infections may develop chronic forms in the future and act as reservoirs contributing to UGS transmission [16–18].

Tests for circulating cathodic antigen (CCA) or circulating anodic antigen (CAA) detection are a rapid and sensitive alternative for identifying active infection and can be implemented in the field; however, they are not available in clinical diagnostic laboratories. As for serological tests, the detection of *Schistosoma-*specific antibodies can determine whether an individual has ever been exposed to the parasite, however, it cannot differentiate between active and past infections. Therefore, its use in areas of high endemicity is limited [14]. On the other hand, molecular techniques, mainly based on PCR protocols, serve as relevant tools for the detection and indirect quantification of parasite DNA due to their high sensitivity and specificity. They also allow the monitoring of the effectiveness of treatment over time. Nevertheless, these techniques require an adequate infrastructure and highly qualified personnel [19–21].

The main problem caused by chronic UGS is damage to the UT. For the accurate diagnosis of these complications, the WHO recommends the use of ultrasonography imaging whenever normal organometric values based on the height and sex of individuals are available [22]. However, this methodology may miss lesions that are undetectable by ultrasound but do cause macrohematuria and/or microhematuria. [23].

The objectives of this study are to assess the usefulness of real-time PCR for the diagnosis of UGS and its correlation with the epidemiological, clinical, and ultrasonographic characteristics of PSAC diagnosed in Cubal, Angola.

## Methods

### Ethics statement

This study was approved by the Ethics Committee of the Angolan Ministry of Health (reference 41/2021). Written informed consent was obtained from the children’s parents/guardians after explaining the purpose of the study, the samples required, and the methods used. All parents voluntarily signed an informed consent form to participate in the study. All participants diagnosed with UGS by microscopy and/or with hematuria were treated with a single dose of 40 mg/kg praziquantel. In some patients diagnosed by real-time PCR, despite attempts to contact them, it was not possible to locate them retrospectively, thus they did not receive treatment.

### Study area and population

A cross-sectional study was conducted with PSAC subjected to parasitological diagnosis of UGS in the Hospital Nossa Senhora da Paz (HNSP) in Cubal (Angola) between February 19 and May 30, 2022. Conventional recruitment of patients could not be carried out in schools, so an appeal was made to participate in places of high social activity, such as markets and churches, and a total of 99 PSACs were randomly selected [24]. The inclusion criteria were: children under five years of age, apparently healthy, and whose caregivers signed the informed consent. The exclusion criteria for urine sample procedures (UDT, macroscopic examination, microscopy examination, and real-time PCR) were defined follows: all urine samples in which the internal control of real-time PCR did not amplify correctly. Therefore, two of the 99 (2/99; 2.02%) participants were discarded in these analyses.

### Epidemiological and clinical data

The following epidemiological and clinical data were collected: gender, age, a height-for-age-based indicator of malnutrition (z-score), hemoglobin value, and UT abnormalities. Malnutrition was categorized based on z-scores: mild, -1.00 to -1.99; moderate, -2.00 to -2.99; and severe, < -3.00. Hemoglobin was measured using the Haemocheck kit (Remedy Healthcare, New Delhi, India), results with hemoglobin values <11 g/dL were defined as low hemoglobin; two groups of patients with low hemoglobin were established according to whether the values were between 7–11 g/dL or <7 g/dL.

All participants were scheduled for UT ultrasonography, which was performed as described by Sánchez Marqués *et al*. [24]. Abnormalities were classified according to the Niamey protocol provided by the WHO for the evaluation of morbidity caused by *S*. *haematobium*, establishing a score from 0 to 32 for each individual, with the score increasing according to the severity of the abnormalities [22].

Parasitological diagnosis of UGS was made based on the microscopic identification of *S*. *haematobium* eggs in the urine sample and/or detection of the genetic material of the parasite by amplification of the *Dra1* sequence by real-time PCR. Patients with positive urine samples by either of these two diagnostic methods were diagnosed with UGS.

### UDT, macroscopic, and microscopic examination

Macrohematuria assessment and UDT were performed as described by Sánchez-Marqués *et al*. [24]. UDT was used to check for microhematuria, proteinuria, and leukocyturia.

Microscopic analysis was performed after filtering the urine through 40 μm nylon filters (Thermo Fisher, Waltham, MA) to detect the presence of and quantify (eggs/10 mL) *S*. *haematobium* eggs [24]. Positive samples were classified according to WHO standards into high or low-intensity infections depending on whether there were more than or less than 50 eggs/10 mL, respectively [3].

Before filtration, an aliquot of the urine sample (1.5 mL) was obtained and stored at -20°C in the HNSP laboratory and sent at room temperature to the Vall d’Hebron University Hospital Department of Microbiology for real-time PCR.

### Molecular analysis

DNA extraction was performed using the NUCLISENS easyMAG automated system (BioMérieux SA, Marcy-l’Étoile, France). The DNA was extracted from 800 μL of urine sample and eluted in 25 μL of elution buffer, according to the manufacturer’s instructions. Eluted DNAs were kept at -20°C until further real-time PCR analysis.

A duplex real-time PCR targeted to *S*. *haematobium* DNA and the human *RNase P* gene was performed [16]. The primers and probes used for each real-time PCR are shown in Table 1. The final conditions in the PCR mixture were 12.5 μL of QuantiTec Multiplex PCR kit (Qiagen, Manchester, UK), 1 μL of human RNase P detection reagent (TaqMan human RNase P detection reagent; Applied Biosystems, Foster City, CA), and 0.4 μL of each primer and probe. The reaction was performed using 5 μL of eluted DNA in a final volume of 25 μL. Amplification was carried out in the CFX real-time PCR detection system (Bio-Rad, Hercules, CA). The cycling conditions were as follows: denaturation at 95°C for 10 min, followed by 50 cycles at 95°C for 15 s and 60°C for 1 min [16].

**Table 1:** Primers and probes used for DNA detection of the different *Schistosoma* species by real-time PCR.

Target	Primers and probe	Final concentration (μM)	Sequence (5´-3´)*	Amplified region	Reference
***S*. *haematobium***	Sh-Fw (forward)	0.16 μM	GATCTCACCTATCAGACGAAAC	*Dra1* repeat unit	[16]
	Sh-Rv (reverse)		TCACAACGATACGACCAAC		
	Sh-P (probe)	0.08 μM	FAM-TGTTGGTGGAAGTGCCTGTTTCGCAA-BHQ1		
***S*. *intercalatum*, *S*. *mansoni*, and *S*. *haematobium***	Ssp48F (forward)	0.50 μM	GGTCTAGATGACTTG ATYGAGATGCT	Internal -transcribed- spacer-2 (ITS2)	[25]
	Ssp124R (reverse)		TCCCGAGCGYGTATAATGTCATTA		
	Ssp78T (probe)	0.20 μM	FAM-TGG GTTGTGCTCGAGTCGTGGC-BHQ		

* Ambiguities: Y = C or T (pyrimidine).

Negative (nuclease-free water) and positive controls (*S*. *haematobium* DNA extracted from the urine of a patient diagnosed with UGS) were included in every run. Urine samples were considered positive and valid when the threshold cycle (Ct) for the *S*. *haematobium* target was <40 and the internal control was efficiently amplified, respectively.

All negative samples from this first analysis were analyzed by a real-time PCR detecting *S*. *intercalatum*, *S*. *mansoni*, and *S*. *haematobium* to rule out the presence of hybrids and other *Schistosoma* spp. [25].

### Statistical analysis

Statistical analyses were performed using the R-UCA package for Windows (version R-UCA3.3.1). Qualitative variables were expressed as absolute frequencies and percentages. Quantitative variables were described as mean and standard deviation (SD) or median and interquartile range (IQR) according to whether their distribution was normal or not, respectively. Pearson’s correlation test and the point biserial correlation test were used to establish the relationship between some pairs of variables. Associations were assessed using the Chi-square dependence test or Fisher’s exact test. The Student’s t-test was used to evaluate whether the mean Ct in the low-intensity infection group was significantly higher than in the high-intensity group. In all cases, the significance level was set at a *p*-value<0.05.

Diagnostic performance parameters for each test (sensitivity, specificity, positive and negative predictive values) were calculated. Cohen’s Kappa coefficient was used to analyze the level of agreement among tests, which was interpreted as follows: slight agreement (0.00–0.20), fair agreement (0.21–0.40), moderate agreement (0.41–0.60), substantial agreement (0.61–0.80), and almost perfect agreement (0.81–1.00).

## Results

### Demographic, clinical, and UT ultrasound characteristics of the study population

Overall, 99 participants were included in this study. The mean age of the children was 3.20 (SD = 1.51) years and 64/99 (64.65%) were male. The mean hemoglobin value was 8.99 (SD = 2.16) g/dL, with a range of values from 4 to 14 g/dL. According to hemoglobin values, 81/99 (81.82%) children had <11 g/dL, of which 65/81 (80.25%) had between 7–11 g/dL and 16/81 (19.75%) had hemoglobin values <7 g/dL. The nutritional status of the children revealed that malnutrition affected 48/99 (48.48%) children, of whom 17/48 (35.42%) were mildly malnourished, 8/48 (16.67%) were moderately malnourished, and 23/48 (47.92%) were severely malnourished. The mean z-score value was -1.28 (SD = 1.59) and values ranged from -4 to 0. Since some participants dropped out of the study, only 73/99 (72.27%) underwent urinary ultrasound, of which 17/73 (23.29%) showed some abnormality. The severity of the lesions presented a mean value of 1.23 (SD = 3.55) and ranged from 0 to 26, according to the established score.

### Urine sample procedures

#### Urine dipstick test

UDT determined that 31/97 (31.96%) samples had microhematuria, 31/97 (31.96%) had leukocyturia, and 18/97 (18.56%) had proteinuria. A macroscopic examination of the urine sample determined that 10/97 (10.31%) samples had visible hematuria.

#### Microscopy examination

A total of 36/97 (37.11%) urine samples were positive by direct microscopy. Egg quantification values determined a median of 7.90 (IQR = 4.00–15.50) eggs/10 mL. High infection values were found in 5/36 (13.89%) samples (median eggs/10 mL = 71.60, IQR = 60.00–182.00 eggs/10 mL) while 31/36 (86.11%) had low infection values (median eggs/10 mL = 9.05, SD = 8.55 eggs/10 mL).

#### Real-time PCR: results and correlation with demographic and clinical data, UGS diagnosis, and morbidity assessment

*S*. *haematobium* DNA was detected in 63/97 (64.95%) children by the real-time PCR assay. The mean Ct value for positive real-time PCR samples was 30.57 (SD = 5.98). Generic real-time PCR results were negative for all samples tested, ruling out the presence of hybrids or species other than *S*. *haematobium*.

Demographic and clinical data, and UDT and macroscopic examination results regarding real-time PCR results are shown in Table 2. A significantly high proportion of positive cases (47/63; 73.44%) were found in children between three and five years of age (*p*-value<0.05). A total of 16/33 (48.48%) children under three years of age were positive, of which 7/16 (43.75%) were under two years of age. Sex, hemoglobin value, and z-score were not significantly related to real-time PCR results (*p*-value>0.05).

**Table 2:** Demographic, clinical, and diagnostic data according to real-time PCR diagnosis.

				Real-time PCR positive (n = 63) N (%)	Real-time PCR negative (n = 34) N (%)	*p*-value
**Demographic data**		Age	Age (years)*	3.43 (1.32)	2.83 (1.76)	<0.05
			0–2.99 years (n = 33)	16 (48.48)	17 (51.52)	<0.05
			3–5 years (n = 64)	47 (73.44)	17 (26.56)	
		Gender	Female (n = 34)	20 (58.80)	14 (41.20)	>0.05
			Male (n = 63)	43 (68.30)	20 (31.70)	
**Clinical data**		Hemoglobin (g/dL)*		9.10 (2.21)	8.88 (1.95)	>0.05
		Z-score *		-1.29 (1.56)	-1.34 (1.66)	>0.05
**Diagnosis of UGS**	Microscopy	Positives		33 (52.40)	3 (8.80)	<0.001
		Quantification	High intensity (n = 5)	4 (80)	1 (20)	
			Low intensity (n = 31)	29 (93.55)	2 (6.45)	
**Morbidity assessment**	Visual observation	Macrohematuria		9 (14.20)	1 (2.90)	>0.05
	Urine dipstick	Microhematuria		27 (42.90)	4 (11.80)	<0.01
		Leukocyturia		24 (38.10)	7 (20.60)	<0.05
		Proteinuria		16 (25.40)	2 (5.90)	<0.01
	Urinary ultrasound	Injuries		14 (32.60)	3 (10.70)	<0.05
		Score *		1.40 (2.44)	1.07 (4.91)	>0.05

*Results are expressed as the mean value and standard deviation (SD).

In addition, it was observed that the proportion of children positive by real-time PCR with UT abnormalities (32.60%; 14/63) was higher than those with a negative result by real-time PCR with UT abnormalities (10.70%; 3/34) (*p*-value<0.05). The mean value of lesion severity was higher in real-time PCR-positive participants (mean = 1.4; SD = 2.44) than in negative ones (mean = 1.07; SD = 4.91), however, no significant correlation was observed (*p*-value>0.05) (Table 2).

#### Comparative performance of microscopy and real-time PCR

Parasitological diagnosis of UGS was considered by at least one of the two diagnostic methods used (microscopy and/or real-time PCR), showing a total of positive diagnoses in 68.04% (66/97) of patients. Using the total parasitological diagnosis as a reference, we observed that the sensitivity of the real-time PCR technique was higher (94.45%) than that of microscopy (54.54%) (Table 3).

**Table 3:** Comparative performance of UGS diagnostic techniques taking parasitological diagnosis as the reference method (n = 97).

		**UGS** * **N**		**Sensibility** **(CI 95%)** *	**Specificity** **(CI 95%)** *	**PPV** * **(CI 95%)** *	**NPV** * **(CI 95%)** *
		+	-				
**Real-time PCR**	+	63	0	95.45 (86.44–98.82)	100.00 (86.27–100.00)	100.00 (92.84–100.00)	91.18 (75.19–97.69)
	-	3	31				
**Microscopy**	+	36	0	54.55 (41.88–66.68)	100.00 (86.27–100.00)	100.00 (87.99–100.00)	50.82 (37.83–63.71)
	-	30	31				

*UGS: urogenital schistosomiasis, CI: confidence interval, PPV: positive predictive value, NPV: negative predictive value.

In contrast, when using microscopy as the reference method, we observed that the sensitivity of real-time PCR was slightly lower (91.67%), with a specificity of 50.82%. The kappa value between the two techniques of 0.37 showed a fair concordance (Table 4).

**Table 4:** Comparative performance of UGS diagnostic techniques taking microscopy as the reference method (n = 97).

		Microscopy N		Sensibility (CI 95%)*	Specificity (CI 95%)*	PPV* (CI 95%)*	NPV* (CI 95%)*	Kappa (CI 95%)*
		+	-					
**Real-time PCR**	+	33	30	91.67 (76.41–97.82)	50.82 (37.83–63.71)	52.38 (39.51–64.96)	91.18 (75.19–97.69)	0.37 (0.19–0.54)
	-	3	31					

*CI: confidence interval, PPV: positive predictive value, NPV: negative predictive value.

In 33/97 (34.02%) samples, the results were discrepant between real-time PCR and microscopy: 30/33 (90.91%) were positive by real-time PCR and negative by microscopy, and the remaining three (3/33, 9.09%) were positive by microscopy and negative by real-time PCR (Table 4). Of the three latter samples, two of them showed a low intensity of infection according to the microscopic examination (0.8 and 3.0 eggs/10 mL of urine), however, the other showed a high intensity of infection (460 eggs/10 mL of urine). Dilutions of the latter sample were performed in PBS (dilution factors 1:2, 1:5, 1:10, and 1:20); nevertheless, the real-time PCR result remained negative.

### Measurement of the intensity of infection

**Fig 1:**
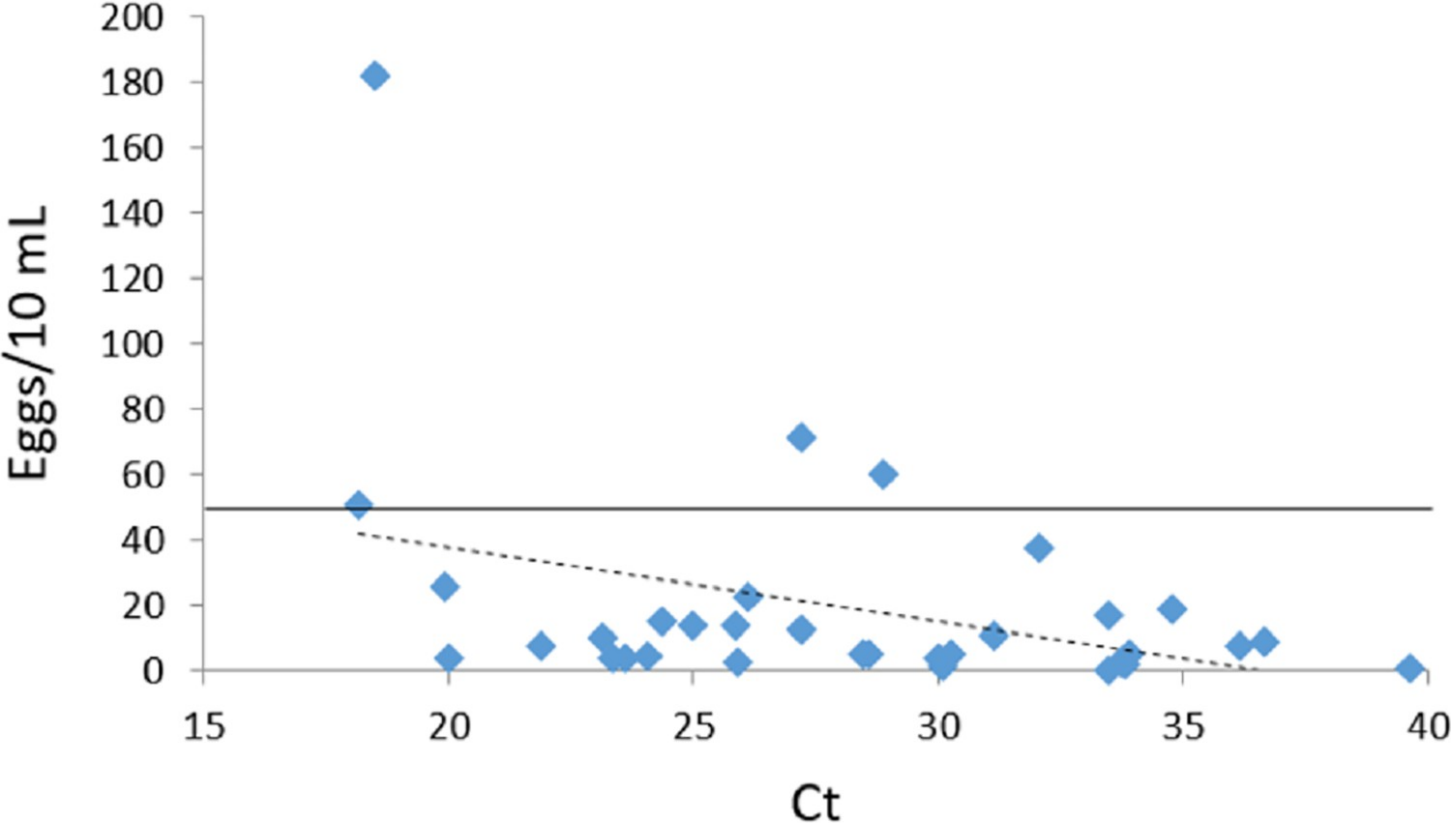
Quantification of positive samples by both microscopy and real-time PCR. Microscopy results are expressed as eggs/10 mL of urine and real-time PCR results as Ct values. Each dot represents one of the participants who were positive by microscopy and real-time PCR (n = 33). The dashed line marks the linear relationship between the two variables. The solid line marks the boundary between high (>50 eggs/10 mL) and low-intensity infection (<50 eggs/10 mL).

The comparison between both techniques exhibited a remarkable correlation between the Ct value and the number of eggs/10 mL (r = 0.38; *p*-value<0.05) (Fig 1). In the high-intensity group, 4/5 (80%) patients were positive by real-time PCR and in the low-intensity group, 29/31 (93.55%) were positive by real-time PCR (Table 2). The mean Ct value was significantly higher in the low-intensity infection group (mean Ct = 28.85, SD = 5.30 Ct) compared with the high-intensity infection group (mean Ct = 23.18, SD = 5.64 Ct) (*p*-value<0.05).

## Discussion

*S*. *haematobium* egg detection by urine microscopy is currently the gold standard method for the diagnosis of UGS despite its low sensitivity [15]. As expected, in our study, the number of UGS cases in PSAC determined by real-time PCR (64.95%) was higher than those obtained by microscopy (37.11%). These results agree with those published in other studies, where the percentage of UGS positives was higher by PCR than by microscopy [16,26].

In this study, we found that the sensitivity of real-time PCR for the detection of *S*. *haematobium* DNA (95.45%) was almost twice that of egg detection by microscopy (54.50%). Our results agree with two previous studies conducted in a SAC population in Kenya and in adults in Nigeria, where the sensitivity of real-time PCR (92% and 100%, respectively) was significantly higher than that of microscopy (31% and 70.1%, respectively) [19,26]. The number of missed *S*. *haematobium* infections in PSAC when only microscopy is used leads to inadequate treatment and further progression of the disease. In addition, these untreated patients act as reservoirs for UGS, promoting its transmission.

The higher sensitivity of real-time PCR makes it a good alternative for UGS diagnostics in PSAC, provided the conditions for its implementation are appropriate. The skilled labor and high cost related to real-time PCR are an inconvenience in resource-limited settings such as Cubal. Therefore, it would be necessary to establish a reference center in endemic areas, capable of performing molecular diagnostics, to carry out screening programs to reveal the true prevalence of UGS, mainly in high-risk populations such as PSAC, and make appropriate public health decisions. On the other hand, other molecular methods such as loop-mediated isothermal amplification have shown higher sensitivity than urine microscopic examination and are potentially applicable in the field [27].

Age was significantly related to the probability of contracting UGS according to real-time PCR results (*p*-value<0.05): a higher percentage of UGS infections by real-time PCR were found in children aged between three and five years compared with children under three years (73.44% vs. 48.48%, respectively), probably due to a higher number of exposures to infested water [28]. These results are consistent with the previous study conducted in Cubal in PSAC by Sánchez-Marqués and another conducted in Nigerian children, where the probability of infection by *S*. *haematobium* is strongly age-dependent [24,29]. On the other hand, other studies provide data in which infection is not significantly related to age [2,30], as there are other risk factors, such as the proximity of the home to infested water sources or the work performed by the children’s caregivers, as it is customary to take them with them during work or household activities [28].

Some studies indicate that gender is a risk factor for *Schistosoma* infection, such that boys are at higher risk of infection than girls [31–34]. However, in our study, in which real-time PCR was used as a diagnostic method, infection was not significantly related to sex, hemoglobin values, or malnutrition scores (*p*-value>0.05).

Parameters determined by UDT (microhematuria, proteinuria, and leukocyturia) and UT images obtained by ultrasonography are widely used to assess the morbidity associated with UGS [35]. In our study, a significant percentage of children with positive real-time PCR results had microhematuria, leukocyturia, and proteinuria (42.9%, 38.1%, and 25.4%, respectively; *p*-value <0.05). Our findings are supported by other studies, in which UDT analysis showed a positive association with egg detection by microscopy [2,24,36]. In this case, our real-time PCR results support the use of UDT as an indirect diagnostic method for UGS in PSAC in high-transmission settings when access to other more sensitive methods is not possible.

According to UT damage, previous studies conducted in Cubal found a correlation between the diagnosis of UGS by microscopy and the severity of lesions observed on urinary ultrasound [24]. In contrast, our real-time PCR results and the severity of lesions were not significantly related (*p*-value>0.05), despite the significant relationship between the presence of UT abnormalities and real-time PCR results (*p*-value<0.05). The high sensitivity of real-time PCR, compared with microscopic examination, allows us to advance the diagnosis before the disease has progressed sufficiently to cause large lesions in the UT.

In a recent study conducted in the PSAC population of Cubal by Sánchez-Marqués using microscopy as the only diagnostic method, a prevalence of 30.2% was obtained [24]. UGS prevalence in PSAC has been previously described in different African countries using urine microscopy as a diagnostic method, ranging from 0.83% to 71.8% [30,37–40]. In contrast, in a study conducted in Zanzibar (Tanzania) using CAA, the prevalence of UGS was 14%, showing a higher positive rate than microscopic examination (5%) and UDT (4%), with higher sensitivity [41]. Therefore, it could be an effective alternative for UGS diagnosis in endemic areas; there are few studies comparing CAA and real-time PCR.

However, when we used real-time PCR as a diagnostic technique in the PSAC population, the number of positive results increased to 64.95% compared with microscopy (37.11%). In a study conducted in Senegal, real-time PCR was used for the diagnosis of UGS in PSAC, obtaining a positivity rate of 12.6%, lower than in our study [42]. Combining the results obtained by microscopy and real-time PCR, our positivity rate was 68.04%. The fact that the positive rate by real-time PCR is almost double that observed by microscopy indicates that the number of cases in which *S*. *haematobium* infection occurs in the first months of life is higher than expected according to previous results [24].

Epidemiologic surveys using only urine microscopy for diagnosis underestimate the true prevalence of UGS in PSAC. This underdiagnosis is a problem when designing control plans and MDA campaigns. The real-time PCR results have demonstrated a high prevalence of infection in this vulnerable population with difficult access to diagnosis. The UT-associated pathology can appear in the first years of life, as observed by Barda *et al*., who found that children could develop UT lesions as early as 6 months after infection [43]. For all these reasons, the inclusion of PSAC including children under two years of age should be considered in the WHO recommendations for MDA campaigns against UGS in high-prevalence areas such as Cubal.

The discrepancies found between real-time PCR and microscopy results were probably due to the higher sensitivity of PCR. However, there were also three samples in which the microscopic examination was positive and real-time PCR negative. Two of these samples corresponded to low-intensity infections, it is possible that conditions during the transport and handling of the urine samples could have led to the degradation of the parasite’s genetic material with the consequent non-detection of its DNA. In contrast, the remaining sample showed a high intensity of infection by microscopy. Excess DNA was ruled out by serial dilutions and, unfortunately, we have no explanation for this discordance.

In this study, we have demonstrated a correlation between parasite density determined by microscopy and Ct values obtained by real-time PCR, as previously described by Keller *et al*. [16]. Nevertheless, real-time PCR only provides an indirect measure that can serve as an indicator of high or low-intensity infection, as established by egg detection via microscopy according to the WHO [3]. One important point is that recent studies have shown that the detection and quantification of *S*. *haematobium* infections by real-time PCR are less affected by day-to-day variations in egg excretion than microscopy. In addition, real-time PCR allows the monitoring of treatment efficacy over time [19–21].

In conclusion, our study has shown that real-time PCR is a highly sensitive method for UGS detection in children under five years of age. In PSAC, real-time PCR, due to its high sensitivity, anticipates the appearance of UT lesions in children under two years of age who, due to their young age, are not yet included in MDA programs. We have also observed that real-time PCR can provide an approximate measure of the intensity of infection, similar to that of egg detection by microscopy. The significant advantages of real-time PCR warrant the need to develop strategies to enable its implementation in the field, despite the technical difficulties.
